# Lateral Intercostal Artery Perforator Flap to Treat Pure Cutaneous Recurrence After Breast-Conserving Surgery: A Case Report

**DOI:** 10.7759/cureus.34364

**Published:** 2023-01-30

**Authors:** Gabriella Savage, E-Ern Ian Ng, Eric Donaldson

**Affiliations:** 1 Surgical Department, Darling Downs Hospital and Health Service, Toowoomba, AUS

**Keywords:** licap flap, cutaneous recurrence, surveillance, bcs, breast cancer

## Abstract

Pure cutaneous recurrence after breast-conserving surgery is rare and presents a unique challenge to clinicians. Some carefully selected patients may be amenable to further breast-conserving therapy. We present the case of a 45-year-old female with a cutaneous recurrence of previously treated right breast cancer along the operative scar in the upper outer quadrant. The patient underwent a further wide local excision with lateral intercostal artery perforator flap with a skin paddle reconstruction. We achieved volume replacement with this technique, disease control, and a pleasing cosmetic result.

## Introduction

Breast-conserving surgery (BCS) is a well-established treatment modality for patients with early breast cancer and offers good cosmesis, low rates of recurrence, and survival outcomes comparable to mastectomy [1,2]. In patients who decline adjuvant treatment, locoregional recurrence rates are higher [2]. While there is limited data on the outcomes of patients who have recurrence treated with further BCS, select reports show no detriment to disease-free survival compared to patients who underwent a completion mastectomy [3,4]. Patients who undergo further BCS must be carefully selected, have favorable tumor biology, and be involved in multidisciplinary planning [5]. Purely cutaneous breast cancer recurrence is rare and presents a unique surgical challenge in patients who decline completion mastectomy. However, oncoplastic volume replacement surgery offers patients excellent treatment outcomes and cosmetic results. The following case outlines the use of a modified lateral intercostal artery perforator (LICAP) flap technique to treat a patient with a cutaneous recurrence of breast cancer.

## Case presentation

**Figure 1:**
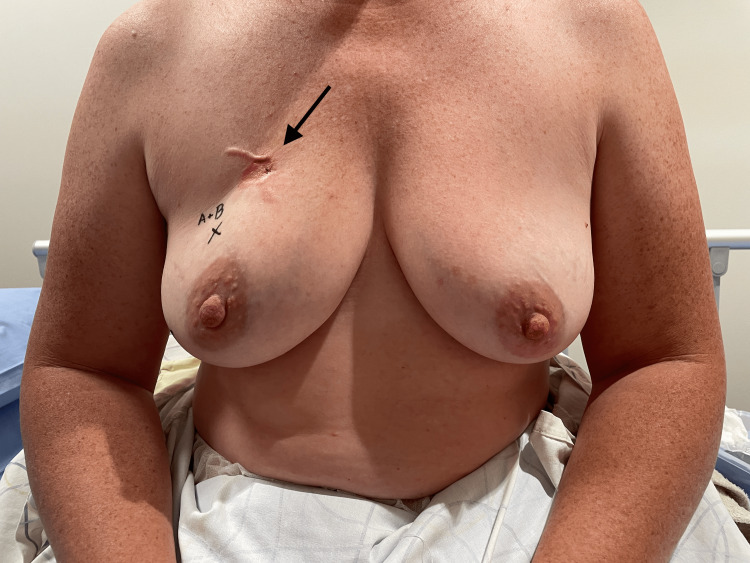
Preoperative imaging. Arrow indicates the recurrent lesion.

**Figure 2:**
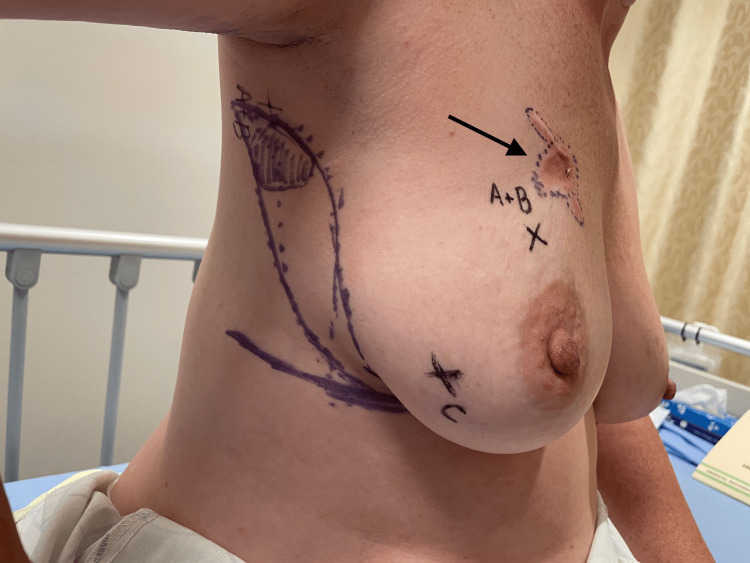
Preoperative imaging. Arrow indicates the recurrent lesion.

**Figure 3:**
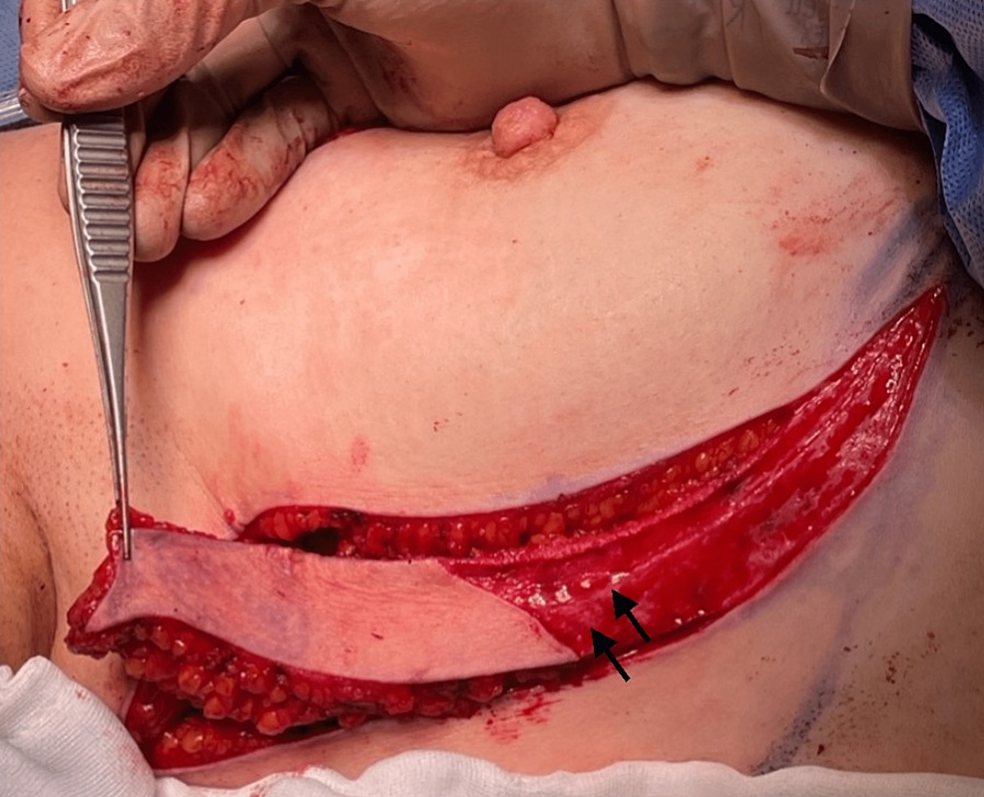
Intraoperative mobilization of the lateral intercostal artery perforator flap with skin paddle preserved. Arrows indicate where the lateral intercostal artery gives perforators to the flap.

**Figure 4:**
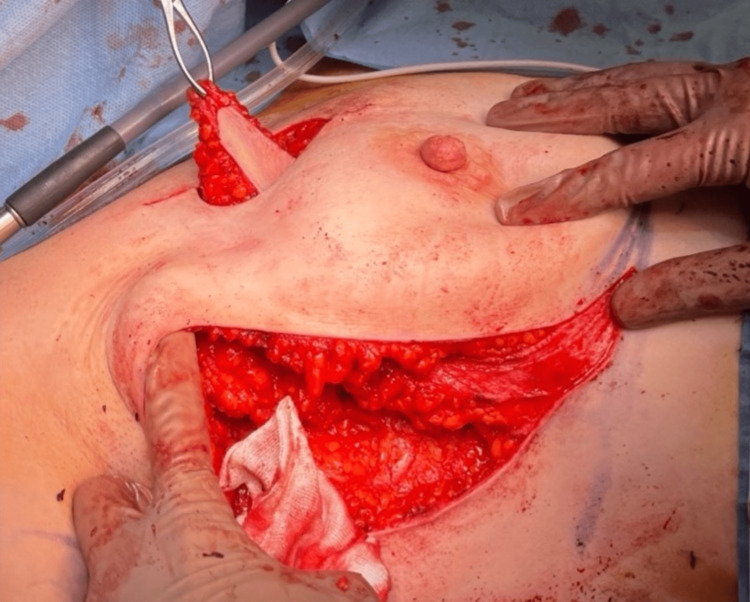
Intraoperative mobilization of the lateral intercostal artery perforator flap with skin paddle preserved.

**Figure 5:**
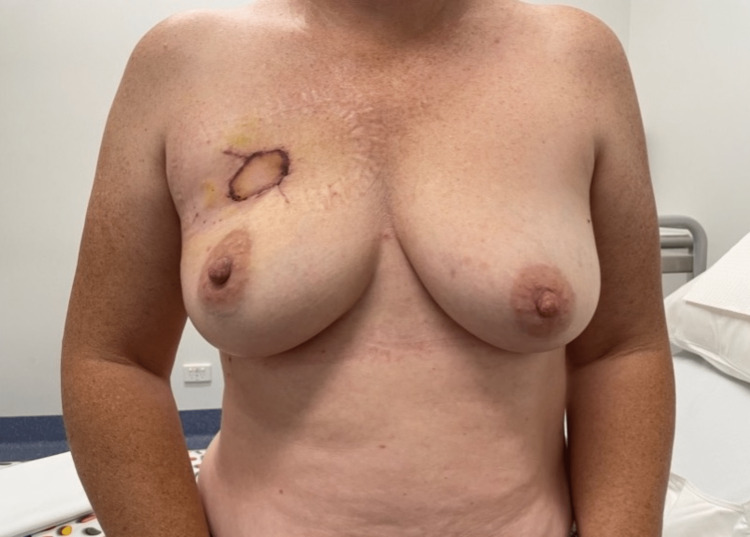
One week postoperatively.

**Figure 6:**
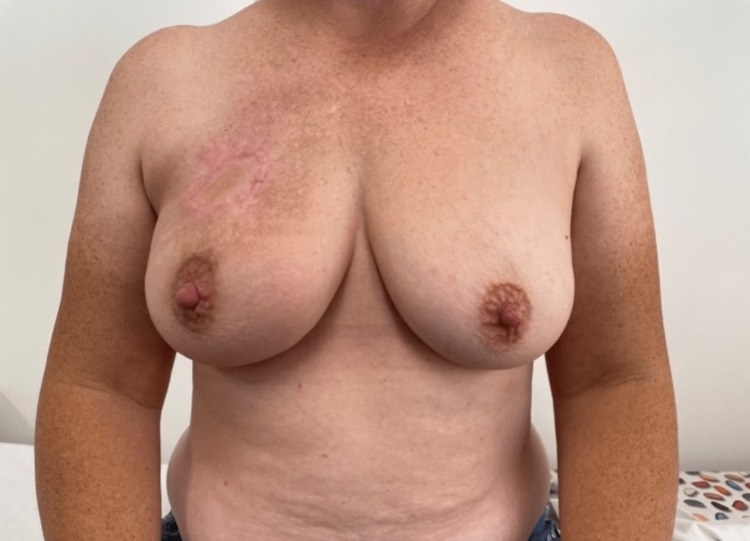
Six months postoperatively.

A 45-year-old female was diagnosed with a cutaneous breast cancer recurrence in the upper outer quadrant of her right breast approximately 10 years after a lumpectomy for early breast cancer (Figures 1, 2). At the time of her initial diagnosis, she underwent BCS but declined adjuvant radiation treatment. The patient had a history of smoking but no other medical or family history. She presented with a new keloid scar at her previous surgical site and had no clinically apparent lymphadenopathy. A subsequent core biopsy confirmed an invasive ductal carcinoma (hormone positive, human epidermal growth factor receptor 2 negative), with the recurrence confined to the skin. Staging CT imaging did not detect any distant metastatic disease. On review by her surgeon, she declined a completion mastectomy and wanted to explore options for further BCS. To achieve volume replacement and preserve breast symmetry, we utilized a modified LICAP flap with a skin paddle to fill the cutaneous defect. This achieved a complete resection of the tumor and an excellent cosmetic result for the patient, who subsequently underwent radiation treatment (declining adjuvant chemotherapy). Previous research has described a modified LICAP flap situated along the lateral mammary fold. This employs a novel technique by leaving a skin paddle at the distal aspect of the LICAP flap (Figures 3, 4). In this case, this technique was used to fill the cutaneous defect resulting from the excision of the recurrent tumor and avoiding contour deformities and asymmetry. The volume of the flap allowed the parenchymal defect from the patient’s previous excision to be corrected. Our patient achieved clear oncological margins and a very good overall cosmetic result with good symmetry (Figures 5, 6).

## Discussion

BCS with adjuvant treatment achieves survival rates equivalent to those seen in patients treated with a mastectomy, while also achieving favorable cosmetic outcomes and low rates of locoregional recurrence [1]. The risk of ipsilateral recurrence following BCS ranges from 2-10% with a median onset of three to four years [6]. Risk factors for recurrence include younger age at diagnosis, large tumor size, high tumor grade, presence of lymphovascular invasion, and absence of hormone receptors [5]. In addition, patients who decline radiation treatment following BCS are at an increased risk of developing locoregional recurrence [2]. Greater than 80% of tumors that recur are morphologically invasive. Detection is through the discovery of a new lesion on clinical examination or surveillance imaging [2,7].

A completion mastectomy is usually the treatment modality of choice for ipsilateral breast cancer recurrence. More recently, further BCS has emerged as a feasible option in select patients. Alpert et al. published outcomes of completion mastectomy versus further BCS in 146 patients with ipsilateral breast cancer recurrence [3]. After a median follow-up time of 13.8 years, there was no statistically significant difference between either treatment approach. Salvadori et al. came to a similar conclusion reporting robust survival rates in patients who underwent further BCS for recurrence compared to those who underwent mastectomy [7]. Komoike et al. also reported no significant difference in survival rates for 41 patients treated with salvage BCS who were followed up for five years [4].

Oncoplastic techniques have continued to develop and offer patients good oncological outcomes with excellent cosmesis. In 1979, anatomical studies by Kerrigan and Daniel provided a sound understanding of intercostal vessels and skin flaps in the surgical repair of large trunk defects [8]. Focusing on cadaveric dissections, the authors identified the precise branching patterns of the lower intercostal neurovascular bundles and determined multiple ways in which versatile skin flaps could be created and surgical techniques could be improved. Badran et al. explored this further developing the technique to fashion a free or island flap using a single intercostal neurovascular bundle and sparing abdominal musculature [9].

Hamdi et al. elaborated on the utility of intercostal perforator (ICAP) flaps in breast reconstructive surgery in 2006 [10]. The concept of perforator flaps, described by Kroll and Rosenfield and Koshima et al., affirmed that any flap could be created out of tissue with an adequate blood supply that could be successfully isolated. This avoids the need to rely solely on larger, named vessels [11,12]. Hamdi et al. described the use of the LICAP flap in breast reconstruction using vessels located over the anterior border of the latissimus dorsi to repair defects in the lateral quadrant of the breast. They concluded that using the ICAP flaps provided an adequate cover of defects without sacrificing muscle [10].

## Conclusions

The modified LICAP flap with a skin paddle is a novel way to replace volume and associated cutaneous defects in the breast tissue. Examples include cutaneous recurrence of breast cancer, as described, in patients who have had previous scar retraction or even skin necrosis where skin and volume replacement is required. It is not limited to just cutaneous recurrences, which are rare in themselves. The procedure offers both excellent oncological margins and cosmetic results and is suitable in carefully selected patients who participate in multidisciplinary treatment planning.
